# A Novel Post-Operative ALRI Model Accurately Predicts Clinical Outcomes of Resected Hepatocellular Carcinoma Patients

**DOI:** 10.3389/fonc.2021.665497

**Published:** 2021-07-06

**Authors:** Minjun Liao, Jiarun Sun, Qifan Zhang, Cuirong Tang, Yuchen Zhou, Mingrong Cao, Tao Chen, Chengguang Hu, Junxiong Yu, Yangda Song, Meng Li, Weijia Liao, Yuanping Zhou

**Affiliations:** ^1^ Guangdong Provincial Key Laboratory of Gastroenterology, Department of Gastroenterology and Hepatology Unit, Nanfang Hospital, Southern Medical University, Guangzhou, China; ^2^ Department of Hepatobiliary Surgery, Nanfang Hospital, Southern Medical University, Guangzhou, China; ^3^ Department of General Surgery, Integrated Hospital of Traditional Chinese Medicine, Southern Medical University, Guangzhou, China; ^4^ Department of Hepatobiliary Surgery, the First Affiliated Hospital of Jinan University, Guangzhou, China; ^5^ Department of Hepatobiliary Surgery, Sun Yat-Sen Memorial Hospital, Sun Yat-Sen University, Guangzhou, China; ^6^ Laboratory of Hepatobiliary and Pancreatic Surgery, Affiliated Hospital of Guilin Medical University, Guilin, China

**Keywords:** Hepatocellular carcinoma, ALRI, biomarker, post-operative, prognosis

## Abstract

**Background:**

Hepatocellular carcinoma (HCC) is one of the leading malignant tumors worldwide. Prognosis and long-term survival of HCC remain unsatisfactory, even after radical resection, and many non-invasive predictors have been explored for post-operative patients. Most prognostic prediction models were based on preoperative clinical characteristics and pathological findings. This study aimed to investigate the prognostic value of a newly constructed nomogram, which incorporated post-operative aspartate aminotransferase to lymphocyte ratio index (ALRI).

**Methods:**

A total of 771 HCC patients underwent radical resection from three medical centers were enrolled and grouped into the training cohort (n = 416) and validation cohort (n = 355). Prognostic prediction potential of ALRI was assessed by receiver operating curve (ROC) analysis. The Cox regression model was used to identify independent prognostic factors. Nomograms for overall survival (OS) and disease-free survival (DFS) were constructed and further validated externally.

**Results:**

The ROC analysis ranked ALRI as the most effective prediction marker for resected HCC patients, with the cut-off value determined at 22.6. Higher ALRI level positively correlated with larger tumor size, higher tumor node metastasis (TNM) stage, and inversely with lower albumin level and shorter OS and DFS. Nomograms for OS and DFS were capable of discriminating HCC patients into different risk-groups.

**Conclusions:**

Post-operative ALRI was of prediction value for HCC prognosis. This novel nomogram may categorize HCC patients into different risk groups, and offer individualized surveillance reference for post-operative patients.

## Introduction

Hepatocellular carcinoma (HCC) is the sixth most common malignant cancer, and the fourth leading cause of cancer-related death in the world (1). Liver cancer results from multiple factors, chief among them is chronic hepatitis B virus (HBV) infection (2, 3), which is endemic in east-Asian and sub-Saharan African regions (4), where 85% of liver cancer incidence occurred (5). Globally, 248 million people are chronically infected with HBV, and a significant portion of them may develop into cirrhosis and liver cancer in the absence of early detection and effective treatments (6). Liver cancer patients could benefit from several radical treatments including surgical resection, regional ablation, and liver transplantation (7). To date, curative resection remains to be a first choice if cancer lesion deemed resectable. But recurrence or distant metastasis were reported in 60–70% patients within 5 years after surgery (8, 9). It is critical that HCC patients participate in post-operative follow-ups and monitorings.

New tumor biomarkers were identified to detect liver cancer in early-stage (10–12), and various prognostic models that aim to predict post-operative prognosis for liver cancer have been developed, such as aspartate aminotransferase to lymphocyte ratio index (ALRI) reported in our previous study and other studies (13, 14); moreover, systemic immune-inflammation index (SII) and neutrophil to lymphocyte ratio (NLR) were reported frequently in many studies (15–19). However, these models mainly used the preoperative data and few incorporated long-term follow-up results. The importance of long-term follow up data in predicting prognosis lies in the fact that clinical outcome of each patient can be determined through early detection of recurrent cancer or metastasis and new treatment options may be selected during the follow-ups. The prognosis prediction mainly based on preoperative factors is insufficient, while accumulated data and results from postoperative surveillance may indicate how HCC patients generally further develop after surgery. Among the indices mentioned above, which one of them could tell prognosis of patients when applying the post-operative data remains unstudied; and whether we could made more accurate prognosis prediction or not remains a challenging task. In this study, we made further investigation into the ALRI index using hematological examination results obtained 2 months after operation, as well as further evaluation of the underlying prediction potency of the novel nomogram which incorporated post-operative ALRI.

## Materials and Methods

### Patients Enrollment

**Figure 1:**
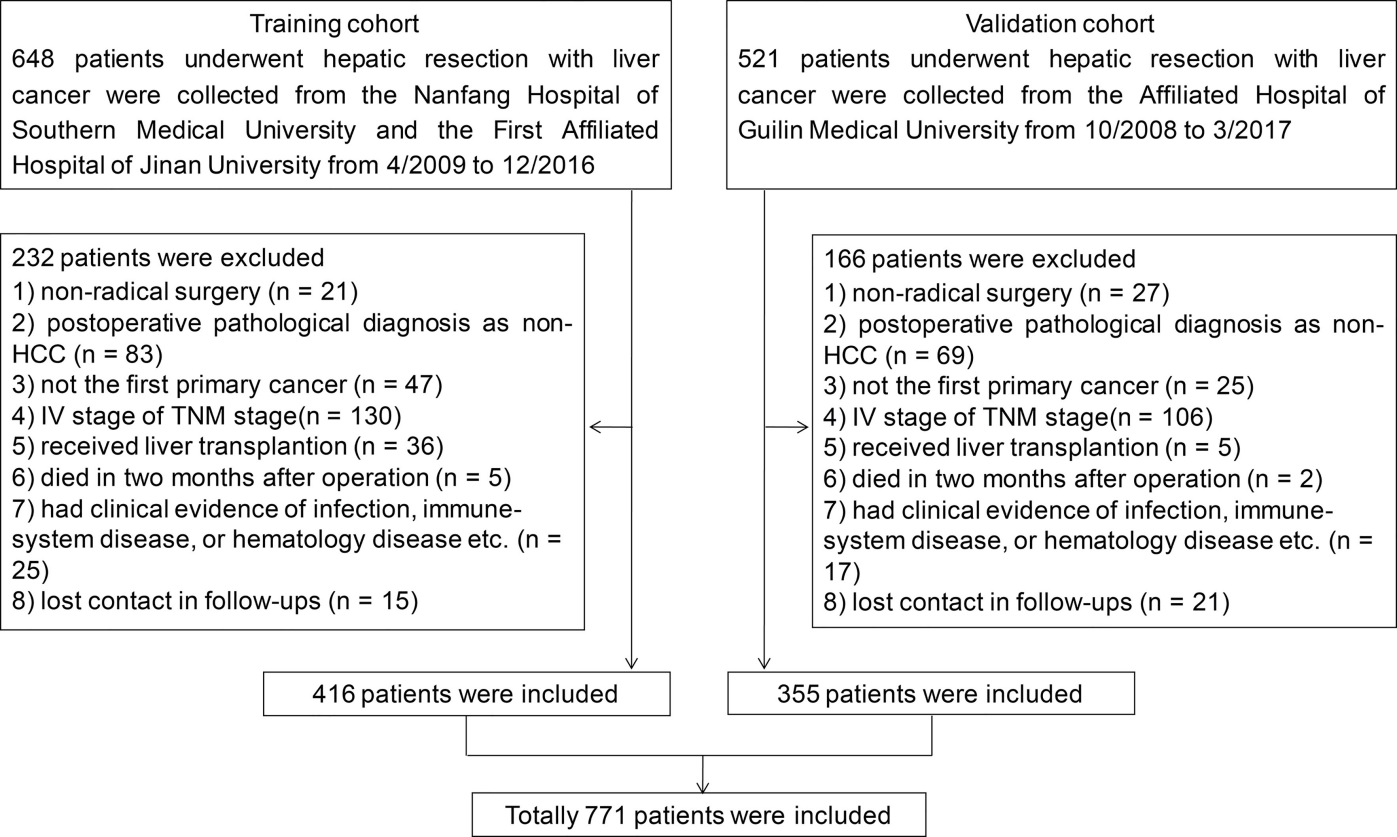
HCC patients’ enrollment flowchart.

A total of 1,169 HCC patients were initially retrospectively analyzed, and 648 patients among them underwent hepatic resection in the Nanfang Hospital, Southern Medical University and the First Affiliated Hospital of Jinan University from April 2009 to December 2016, and the remaining 521 patients received hepatic resection in the Affiliated Hospital of Guilin Medical University from October 2008 through March 2017. The exclusion criteria were as follows: 1) non-radical surgery; 2) postoperative pathological diagnosis as non-HCC; 3) not the first primary cancer; 4) IV stage of TNM stage; 5) received liver transplantation; 6) died in 2 months after operation; 7) with clinical evidence of infection, immune-system diseases, or hematological diseases etc.; 8) lost contact in follow-ups. Finally, 771 patients were eligible for final analyses, 416 from Nanfang Hospital of Southern Medical University and the First Affiliated Hospital of Jinan University as training cohort and 355 patients from the Affiliated Hospital of Guilin Medical University as validation cohort. The flowchart of patient selection is shown in **Figure 1**.

### Clinicopathologic Characteristics of HCC Patients

HCC patients’ baseline information and clinical data were collected, including (1) preoperative demographics and medical history: age, gender, family history, drinking and smoking history, and hepatitis B virus infection history etc.; (2) hematological examination data obtained during follow-up of 2 months after operation: white blood cell (WBC), neutrophil, lymphocyte, and platelet count; albumin, aspartate aminotransferase (AST), alanine aminotransferase (ALT), gamma-glutamyl transpeptidase (GGT), total bilirubin (TBIL), α-fetoprotein (AFP), etc.; (3) the number of tumor, tumor size, Child stage and TNM stage, etc.; (4) pathological lesions of cirrhosis, and recurrence (**Table 1**). We decided to choose hematological examination results of 2 months after operation as the time-point in consideration of the reason that generally this was the first time-point during regular follow-ups. Post-operative ALRI was calculated based on the following formula: (AST value/lymphocyte count) × 10^9^/U, and SII = P × N/L, NLR = N/L, where P, N, and L were the peripheral platelet, neutrophil, and lymphocyte counts, respectively. The study was approved by the research ethics committee of the Affiliated Hospital of Guilin Medical University, Nanfang Hospital of Southern Medical University and the First Affiliated Hospital of Jinan University, and conformed to the Declaration of Helsinki. Written informed consents were obtained from all patients.

**Table 1:** Comparison of clinicopathological characteristics of two groups’ patients.

Parameter	Total patients (n = 771)	Training cohort (Guangzhou)	Validation cohort (Guilin)	*p* value
		(n = 416)	(n = 355)	
Gender: female/male (n)	101/670	57/359	44/311	0.592
Age (years)	50.07 ± 11.41	50.47 ± 11.15	49.61 ± 11.71	0.297
HBsAg: negative/positive (n)	123/648	63/353	60/295	0.507
Family history: absent/present (n)	687/84	371/45	316/39	0.940
Alcohol abuse: absent/present (n)	420/351	238/178	182/173	0.099
Smoking: absent/present (n)	441/330	250/166	191/164	0.078
Cirrhosis: absent/present (n)	58/713	32/384	26/329	0.847
Tumor size (cm)	8.04 ± 4.53	7.90 ± 4.62	8.22 ± 4.45	0.335
Tumor number: single/multiple (n)	543/228	291/125	252/103	0.754
Child stage: A/B (n)	688/83	369/47	319/36	0.605
TNM stage: I/II/III (n)	113/287/371	66/148/202	47/139/169	0.450
Recurrence: absent/present (n)	430/341	224/192	206/149	0.244
Hematology test value 2 months after operation
WBC (×10^9^/L)	6.91 ± 2.65	7.01 ± 2.63	6.80 ± 2.69	0.275
Platelets (×10^9^/L)	194.66 ± 92.33	200.35 ± 98.20	188.57 ± 85.69	0.079
NEUT (×10^9^/L)	4.42 ± 2.37	4.51 ± 2.35	4.33 ± 2.41	0.303
LYMPH (×10^9^/L)	1.63 ± 0.62	1.63 ± 0.66	1.62 ± 0.60	0.849
Albumin (g/L)	36.01 ± 5.99	35.68 ± 6.01	36.45 ± 5.95	0.074
Globulin (g/L)	33.68 ± 6.50	34.01 ± 6.75	33.30 ± 6.19	0.134
TBIL (μmol/L)	16.20 ± 10.78	16.10 ± 10.56	16.32 ± 11.05	0.772
DBIL (μmol/L)	7.50 ± 7.03	7.43 ± 6.82	7.58 ± 7.27	0.778
ALT (U/L)	47.03 ± 42.43	46.05 ± 42.04	48.19 ± 42.93	0.484
AST (U/L)	49.60 ± 42.03	50.81 ± 44.35	48.06 ± 38.89	0.378
GGT (U/L)	120.80 ± 110.74	117.38 ± 108.16	124.90 ± 113.78	0.361
ALP (U/L)	107.12 ± 76.69	105.80 ± 82.52	108.63 ± 68.78	0.618
AFP (ng/ml): median (IQR)	11.32 (6.31–38.95)	10.98 (5.46–32.36)	13.30 (7.50–47.33)	0.511
NLR level	3.13 ± 2.24	3.19 ± 2.20	3.06 ± 2.28	0.408
SII level	613.98 ± 528.02	630.01 ± 521.10	585.76 ± 530.46	0.172
ALRI level	35.71 ± 33.01	36.18 ± 32.63	35.20 ± 33.26	0.690

n, number of patients; HBsAg, hepatitis B surface antigen; TNM, tumor-node-metastasis; WBC, white blood cell; NEUT, neutrophil count; LYMPH, lymphocyte count; TBIL, total bilirubin; DBIL, direct bilirubin; ALT, alanine aminotransferase; AST, aspartate aminotransferase; GGT, Gamma-glutamyl transpeptidase; ALP, alkaline phosphatase; AFP, alpha-fetoprotein; IQR, interquartile range; NLR, neutrophil to lymphocyte ratio; SII, systemic immune-inflammation index; ALRI, AST to lymphocyte ratio index.

### Follow-Ups

All 771 patients were instructed to attend regular follow-up visits after radical resection. Tumor recurrence was monitored by testing serum AFP, hepatic function, ultrasonography, and chest radiography every 2 months for the first 2 years and every 3–6 months thereafter; and CT enhanced scanning and MRI examination were needed when recurrence were suspected during follow-ups. Average follow-up period was 36.7 months (median, 26.0 months; range, 2.0 to 84.0 months). Disease-free survival (DFS) was defined from date of surgery to date of recurrence, metastasis, death, or the last follow-up; and overall survival (OS) was defined from date of surgery to date of death or the last follow-up.

### Statistical Analysis

Continuous variables conforming to Gaussian distribution were expressed as mean ± standard deviation (SD) and the differences were compared using independent sample t-test, and classification factors were identified by Pearson chi-square test or Fisher exact test. All statistical analyses were conducted using SPSS 24.0 (SPSS Inc, Chicago, IL, USA) and R version 4.0.3 (https://www.rproject.org/). The ROC curve guided selecting the optimal cut-off value of post-operative ALRI and was plotted *via* timeROC package. Univariate and multivariate analyses were used to identify the independent prognostic factors for DFS and OS; and the nomogram was built *via* rms package, while the calibration curve was established by the rms package. Decision curve analysis (DCA) was based on the rmda package, and Cox proportional hazards regression model was employed to construct the novel nomogram. The performance of the novel model was evaluated by the calibration curves, and discriminatory ability was assessed by AUC of the ROC curve. Survival curve analyses were performed using the Kaplan-Meier method. Hazard ratios (HR) and 95% confidence intervals (95% CI) were calculated. *P* < 0.05 was considered statistically significant.

## Results

### Baseline and Post-Operative Information of HCC Patients

A total of 771 patients were enrolled in the study. The training cohort and the validation cohort consisted of 416 and 355 patients, respectively. There was no significant difference in HCC patients’ clinical and pathological characteristics between the training and the validation cohorts (*P* > 0.05).

### Determination of the Optimal Cut-Off Value of Post-Operative ALRI

**Figure 2:**
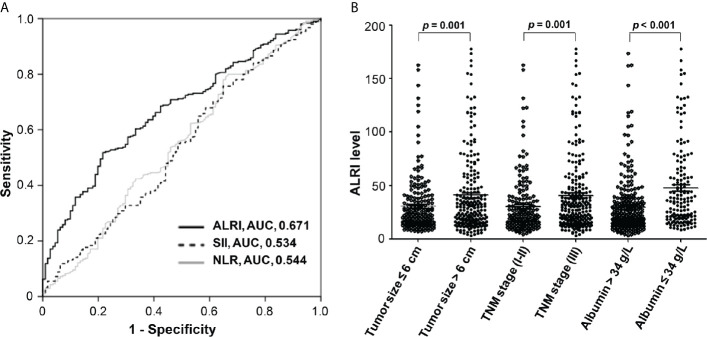
Prognostic prediction value of ALRI for post-operative HCC patients in the training cohort and the comparison of ALRI level in different sub-groups. **(A)** Comparison of prediction performance of ALRI, SII, and NLR using the ROC analyses. **(B)** Comparison of ALRI level in different tumor size, TNM stage, and serum albumin sub-groups.

Receiver operating characteristic (ROC) curve was used to compare post-operative ALRI, SII, and NLR’s prediction potential for post-operative HCC patients. ALRI in both training cohort (**Figure 2A**) and validation cohort (**Figure S1A**) had the largest area under the curve (AUC: 0.671, 95% CI: 0.623–0.716). Sensitivity and specificity reached 61.6 and 67.5%, respectively, when the optimal cut-off value set at 22.6. Furthermore, a comparison of patients’ post-operative ALRI level was made between patients with different tumor size (≤6 or >6 cm), different TNM stage (I-II or III), and different albumin level (≤34 or >34 g/L), and results showed that advanced tumor (tumor size >6 cm, III stage of TNM stage, or lower albumin ≤34 g/L) had higher ALRI level (*P* < 0.05) (**Figures 2B** and **S1B**), suggesting that high ALRI might be associated with poor physical condition of HCC patients, thus leading to poor clinical outcomes.

### Univariate and Multivariate Cox Regression Analyses

In the univariate analysis of training cohort, tumor size (>6 cm), multiple tumor number, TNM stage III, albumin (≤34 g/L), GGT (>45 U/L), ALP (>90 U/L), and ALRI (>22.6) were identified as significant prognostic factors of poor OS and DFS, and their hazard ratio (HR) and 95% confidence interval (95% CI) were shown in **Table 2**. After adjusting other confounding factors, a stepwise multivariate Cox proportional hazards model revealed that tumor size (HR, 1.786; 95% CI, 1.354–2.356; *P* < 0.001), TNM stage (HR, 1.802; 95% CI, 1.420–2.287; *P* < 0.001), albumin (HR, 1.448; 95% CI, 1.126–1.891; *P* = 0.004), and ALRI (HR, 1.872; 95% CI, 1.420–2.467; *P* < 0.001) were identified as independent predictive factors of OS (**Table 2**). Tumor size (HR, 1.479; 95% CI, 1.114–1.965; *P* = 0.007), TNM stage (HR, 1.642; 95% CI, 1.238–2.177; *P* = 0.001), albumin (HR, 1.547; 95% CI, 1.194–2.003; *P* = 0.001), and ALRI (HR, 1.703; 95% CI, 1.339–2.166; *P* < 0.001) were identified as independent predictive factors of DFS (**Table 2**).

**Table 2:** Univariate and multivariate Cox regression analyses of the clinicopathologic characteristics for OS and DFS in training cohort with HCC.

Variable	Univariate analysis			Multivariate analysis		
	HR	95% CI	*p* value	HR	95% CI	*p* value
**Overall survival**						
Sex (male *vs.* female)	1.134	0.810–1.587	0.463			
Age, yeas (≤55 *vs.* 55)	1.339	0.958–1.692	0.055			
HBsAg (positive *vs.* negative)	1.105	0.805–1.516	0.538			
Tumor size, cm (>6 *vs.* ≤6)	2.393	1.839–3.106	<0.001	1.786	1.354–2.356	<0.001
Tumor number (multiple *vs.* single)	1.430	1.125–1.817	0.003			
TNM stage (III *vs.* I-II)	2.593	2.014–3.230	<0.001	1.802	1.420–2.287	<0.001
Recurrence: absent/present (n)	1.020	0.814–1.278	0.863			
Albumin, g/L (≤34 *vs.* >34)	1.615	1.264–2.065	<0.001	1.448	1.126–1.891	0.004
Globulin, g/L (>33 *vs.* ≤33)	1.032	0.823–1.293	0.785			
ALT, U/L (>38 *vs.* ≤38)	1.196	0.953–1.499	0.122			
GGT, U/L (>45 *vs.* ≤45)	1.779	1.283–2.469	<0.001			
ALP, U/L (>90 *vs.* ≤90)	1.456	1.156–1.833	0.001			
AFP, ng/ml (>20 *vs.* ≤20)	1.011	0.766–1.334	0.938			
ALRI level (>22.6 *vs*. ≤22.6)	1.966	1.574–2.534	<0.001	1.872	1.420–2.467	<0.001
**Disease-free survival**						
Sex (male *vs.* female)	1.125	0.804–1.575	0.492			
Age, yeas (≤55 *vs.* >55)	1.320	0.944–1.669	0.105			
HBsAg (positive *vs.* negative)	1.137	0.828–1.560	0.428			
Tumor size, cm (>6 *vs.* ≤6)	2.253	1.783–2.470	<0.001	1.479	1.114–1.965	0.007
Tumor number (multiple *vs.* single)	1.503	1.182–1.911	0.001			
TNM stage (III *vs.* I-II)	2.414	1.894–3.077	<0.001	1.642	1.238–2.177	0.001
Albumin, g/L (≤34 *vs.* >34)	1.719	1.344–2.198	<0.001	1.547	1.194–2.003	0.001
Globulin, g/L (>33 *vs.* ≤33)	1.060	0.846–1.328	0.614			
ALT, U/L (>38 *vs.* ≤38)	1.249	0.996–1.566	0.055			
GGT, U/L (>45 *vs.* ≤45)	1.663	1.199–2.308	0.002			
ALP, U/L (>90 *vs.* ≤90)	1.441	1.144–1.815	0.003			
AFP, ng/ml (>20 *vs.* ≤20)	1.006	0.762–1.328	0.967			
ALRI level (>22.6 *vs.* ≤22.6)	2.081	1.657–2.612	<0.001	1.703	1.339–2.166	<0.001

HR, hazard ratio; CI, confidence interval; HBsAg, hepatitis B surface antigen; TNM, tumor-node-metastasis; ALT, alanine aminotransferase; AST, aspartate aminotransferase; GGT, Gamma-glutamyl transpeptidase; ALP, alkaline phosphatase; AFP, alpha-fetoprotein; ALRI, aspartate aminotransferase to lymphocyte ratio index.

In validation cohort, the results of the univariate and multivariate analyses were very consistent with the training cohort (**Table S1**). In the multivariate analysis, ALRI remained an independent predictor for OS (HR, 1.933; 95% CI, 1.478–2.527; *P* < 0.001) and DFS (HR, 1.701; 95% CI, 1.305–2.218; *P* < 0.001).

### Construction and Evaluations of Prognostic Nomograms for OS and DFS

**Figure 3:**
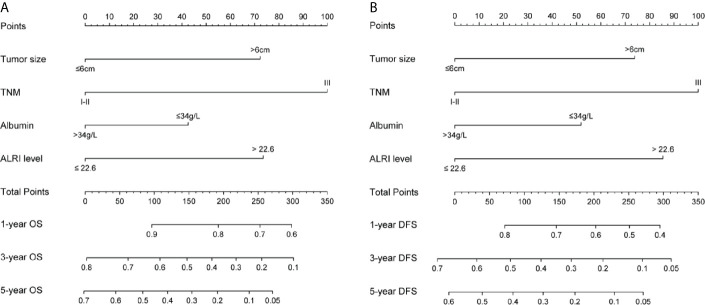
Nomograms for OS and DFS in the training cohort. Sum up the score of each factor, and 1-, 3-, and 5-year OS were determined according to the total score. The 1-, 3-, and 5-year DFS were determined in the same way **(A, B)**.

Tumor size, TNM stage, serum albumin, and ALRI were identified as independent prognostic factors for OS and DFS by univariate and multivariate cox regression analyses mentioned above, and were utilized to construct novel nomograms to predict 1-, 3-, and 5-year OS as well as 1-, 3-, and 5-year DFS for post-operative HCC patients (**Figures 3** and **S2**).

**Figure 4:**
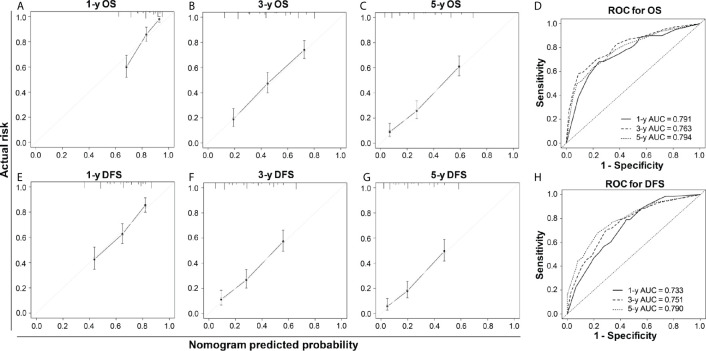
****The calibration curves and ROC curves of 1-, 3-, and 5-year OS **(A–D)** and 1-, 3-, and 5-year DFS **(E–H)** in the training cohort. For the calibration curve, the x-axis was the predicted-survival based on the nomogram, and the y-axis was the actual-survival; the more the predicted line coincided with the diagonal line, the more accurate the prognosis nomogram would be.

Our nomogram showed potential clinical utility as it predicted post-operative survival with C-index of 0.705 (95% CI: 0.661–0.756) for OS and 0.678 (95% CI: 0.631–0.725) for DFS in the training cohort, while, the C-index in validation cohort was 0.711 (95% CI: 0.667–0.763) for OS and 0.666 (95% CI: 0.619–0.714) for DFS. The calibration curves of 1-, 3-, and 5-year OS and 1-, 3-, and 5-year DFS in the training cohort largely coincided with their standard curves, and similar results were observed in validation cohort (**Figures 4A–C, E–G** and **Figures S3A–C, E–G**). In training cohort, the AUC of ROC curves for 1-, 3-, and 5-year OS were 0.791, 0.763, and 0.794 (**Figure 4D**), respectively; and 0.733, 0.751, and 0.790 for 1-, 3-, and 5-year DFS (**Figure 4H**), respectively, achieving more than 70% prediction accuracy for post-operative HCC patients. The AUC of ROC in validation cohort curves were 0.751, 0.810, and 0.783 for 1-, 3-, and 5-year OS, respectively; and 0.723, 0.763, and 0.764 for 1-, 3-, and 5-year DFS, respectively (**Figures S3D, H**), which further validated the predictive performance of the novel nomograms.

### Survival Outcomes

**Figure 5:**
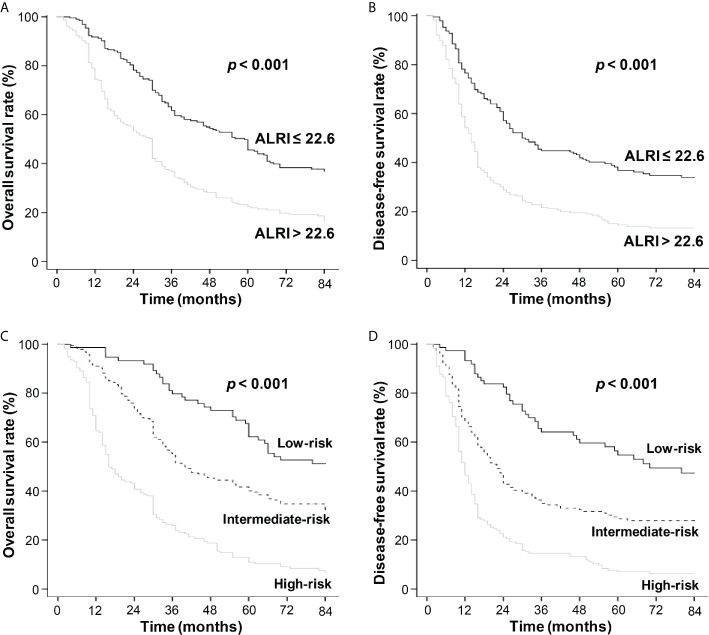
The OS and DFS curves in the training cohort. Kaplan-Meier survival analyses revealed that HCC patients with ALRI > 22.6 had shorter OS and DFS **(A, B)**. The black line refers to ALRI ≤ 22.6 and the gray line refers to ALRI > 22.6. Kaplan-Meier survival analyses of HCC patients in different risk groups **(C, D)**. The black line refers to low-risk group, the dotted line: intermediate-risk group and the gray line: high-risk group.

Kaplan-Meier survival analysis showed that a higher post-operative ALRI value (ALRI > 22.6) was associated with shorter OS and DFS in the training cohort (*P* < 0.001) (**Figures 5A, B**), so was it in the validation cohort (**Figures S4A, B**). The post-operative liver cancer patients were further divided into three different risks’ groups to predict OS and DFS based on their total risk scores calculated by the novel nomogram (patient of score 0–90, 90–190, >190 into the low-, intermediate-, high-risk groups, respectively). The OS and DFS in different risk groups were further analyzed, revealing significant differences in OS and DFS among different risk groups in both training cohort (*P* < 0.001) (**Figures 5C, D**) and validation cohort (*P* < 0.001) (**Figures S4C, D**).

## Discussion

HCC is one of the most aggressive human cancers, which is difficult to cure, as up to 60–70% of HCC patients may experience recurrent cancer and/or metastasis after hepatectomy (8, 9). Recommended management of post-operative HCC patients includes regular monitoring schedule with routine blood and liver function tests, ultrasonography, CT, and MRI examinations. Continuous efforts to identify new tumor biomarkers may help detect early-stage liver cancer, facilitating early intervention and improving clinical outcomes (10–12).

There are several noninvasive and low-cost prognostic predictive models including ALRI (13, 14), NLR, and SII (15–19), which mainly utilize preoperative parameters such as baseline parameters or clinical information collected before surgery, and their performance was relatively satisfactory. However, in order to improve the prediction with those markers, we incorporated the postoperative data to evaluate these predictors in this study.

Distinct outcomes have been clinically noted among HCC patients who shared many similarities including age, gender, tumor size, pathological stage, or laboratory findings. Some patients may achieve up to 10 years of disease-free survival (DFS), while other patients have recurrent cancer 1~2 years after resection, suggesting post-operative conditions likely represent key factors determining different outcomes. We found the study that investigated the influence of post-operative inflammation scores for prognosis in HCC patients after surgery (16). Surprisingly in our pilot study, we found that, NLR and SII based on hematologic findings extracted 2 months after radical resection had unfavorable predictive utility; but ALRI appeared to accurately indicate patients’ physical conditions after surgery and provided as a useful measurement that may offer reference to post-operative treatments.

In some studies, the models applying data of a specific time-point after surgery remained to be effective predictors of HCC prognosis, such as ALBI grade at the first year after resection (20), AFP response (change of AFP before and 1 week after hepatectomy) (21), daily decrease of post-operative AFP (22), postoperative serum osteopontin level (23), etc. In this study, we further investigated the postoperative ALRI model using hematologic findings extracted 2 months after radical resection, which more accurately predicted patients’ physical condition after surgery.

Systematic inflammation is associated with cancer progression by promoting angiogenesis (24), suppressing cell apoptosis and facilitating cancer invasion (25). Several prognostic models have incorporated inflammatory markers, such as lymphocyte counting and AST level. Lymphocytes are crucial in surveillance and suppression of cancer occurrence, growth, and migration (26). The amount of peripheral and infiltrating lymphocytes reflects the intensity of anti-cancer response a cancer patient can assemble (27). Serum AST, released by destructed hepatocytes, is a sensitive and reliable indicator for the extent of liver injury (28, 29). Therefore, ALRI using lymphocyte count and AST level, as reported, had predictive value of clinical outcomes for post-operative HCC patients (13, 14). In addition, serum albumin level represents the functional capacity of liver. Serum albumin level reduced when an injured liver deteriorated into the decompensated state. Clearly, tumor burden negatively impact post-operative prognosis as larger tumor size and advanced tumor are associated with poorer immunological function of patients (30–32).

Guided by the above reasoning, we constructed the novel survival nomograms for OS and DFS to predict outcomes of post-operative HCC patients. Four independent factors were incorporated in this nomogram: tumor size, TNM stage, post-operative serum albumin level, and post-operative ALRI value. Kaplan-Meier survival analyses showed that the nomograms performed well in categorizing HCC patients into different risk groups, and high-risk group had the worst OS and DFS (*P* < 0.001). This new nomogram containing ALRI also showed satisfactory prediction capacity and may bring reference value to post-operative follow-ups and monitorings.

There are limitations in this study. First, this was a retrospective study that may carry inherent bias in enrollment. Second, we didn’t include data beyond 2-month after surgery. Further evaluation of the predictive results of later time-points is required. Third, most of our patients were hepatitis B virus infected. Therefore, future prospective studies that enroll larger sample size of post-operative HCC patients from multiple centers, with different etiologies, may provide further validation of our findings in this study.

## Data Availability Statement

The original data was not available now. Requests to access the datasets should be directed to (liaoweijia288@163.com; yuanpingzhou@163.com).

## Ethics Statement

The studies involving human participants were reviewed and approved by the research ethics committee of the Affiliated Hospital of Guilin Medical University, Nanfang Hospital of Southern Medical University and the First Affiliated Hospital of Jinan University. The patients/participants provided their written informed consent to participate in this study.

## Author Contributions

YPZ and WJL put forward the ideas of this article. MJL, JRS and QFZ wrote this article. CRT, YDS and ML help revise this manuscript. YCZ, MRC and TC helped the collection of data. CGH and JXY conducted the analysis of data. All authors contributed to the article and approved the submitted version.

## Funding

This work was supported in part by the grants from National Natural Science Foundation of China (No. 81772923), and the Science and Technology Planning Project of Guilin (No. 20190218-1).

## Conflict of Interest

The authors declare that the research was conducted in the absence of any commercial or financial relationships that could be construed as a potential conflict of interest.

## Abbreviations

HCC, hepatocellular carcinoma; AST, aspartate aminotransferase; ALRI, aspartate aminotransferase to lymphocyte ratio index; SII, systemic immune-inflammation index; NLR, neutrophil to lymphocyte ratio; ROC, receiver operating characteristic; OS, overall survival; DFS, disease-free survival; TNM, tumor node metastasis; HR, hazard ratios.
