# Supplementary material for: A Novel Post-Operative ALRI Model Accurately Predicts Clinical Outcomes of Resected Hepatocellular Carcinoma Patients
Source: Front Oncol. 2021 Jul 6;11:665497. doi: 10.3389/fonc.2021.665497 (PMC8290124; doi:10.3389/fonc.2021.665497)
Supplement: Supplementary file 1 [file DataSheet_1.docx]

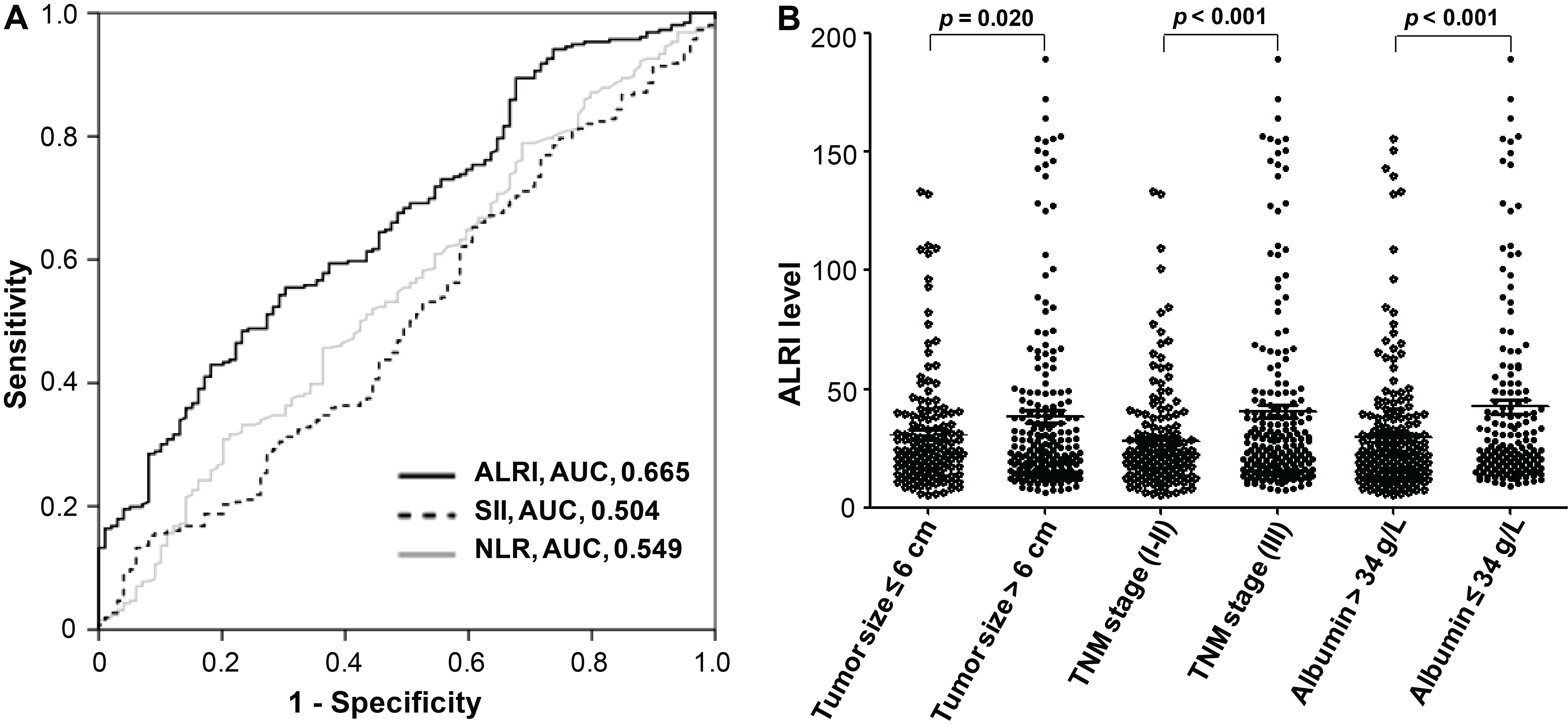


Fig S1. Prognostic prediction value of ALRI for HCC patients after radical resection in the validation cohort, and comparison of ALRI level in different sub-groups. (A) Comparison of predictive performance of ALRI, SII and NLR using the ROC analyses. (B) Comparison of ALRI level in different tumor size, TNM stage and serum albumin sub-groups.


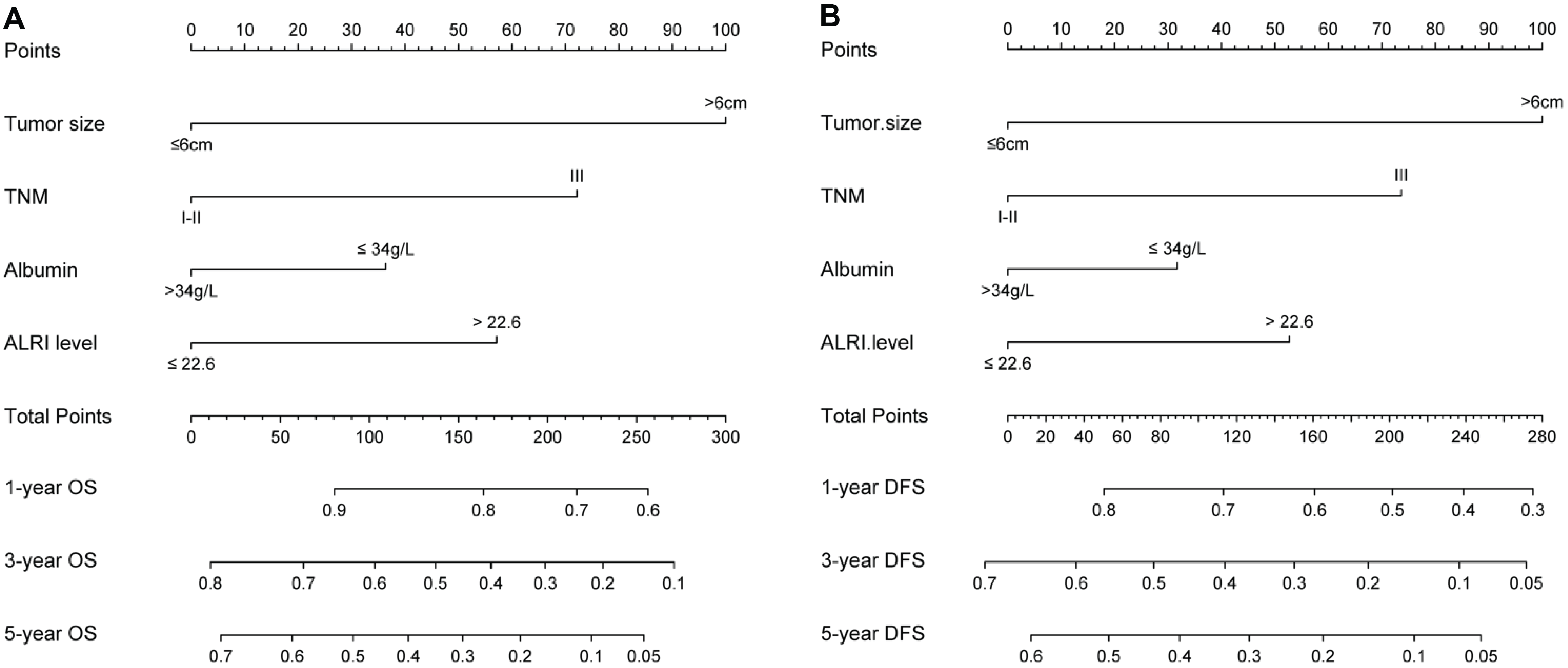


Fig S2. Nomograms for OS and DFS in the validation cohort. Sum up the score of each factor, and 1-, 3- and 5-year OS were determined according to the total score. 1-, 3- and 5-year DFS were determined in the same way (A and B).


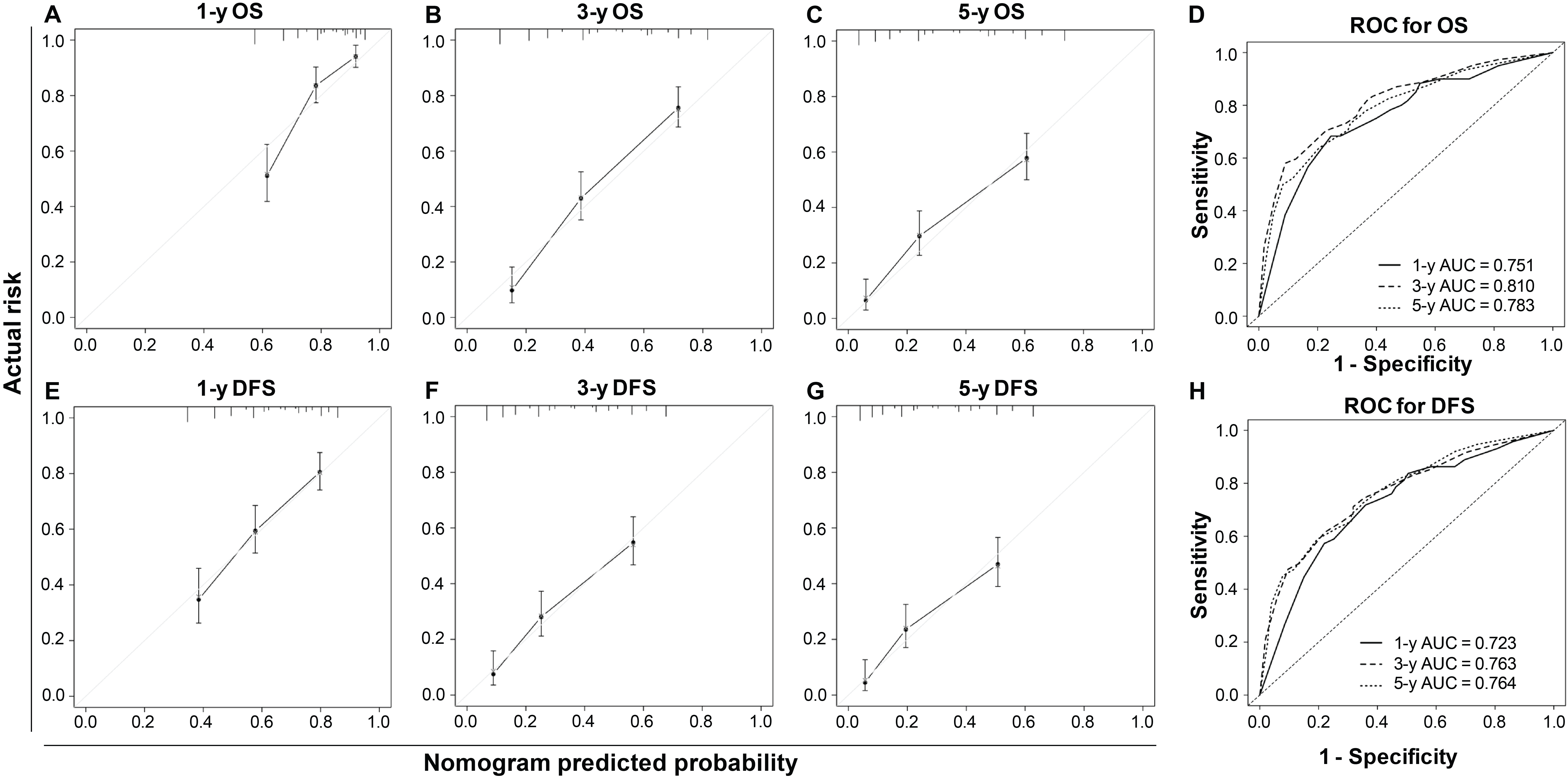


Fig S3. The calibration curves and ROC curves of 1-, 3- and 5-year OS (A-D) and 1-, 3- and 5-year DFS (E-H) in the validation cohort. For the calibration curve, the x-axis was the predicted-survival based on the nomogram, and the y-axis was the actual-survival; the more the predicted line coincided with the diagonal line, the more accurate the prognosis nomogram would be.


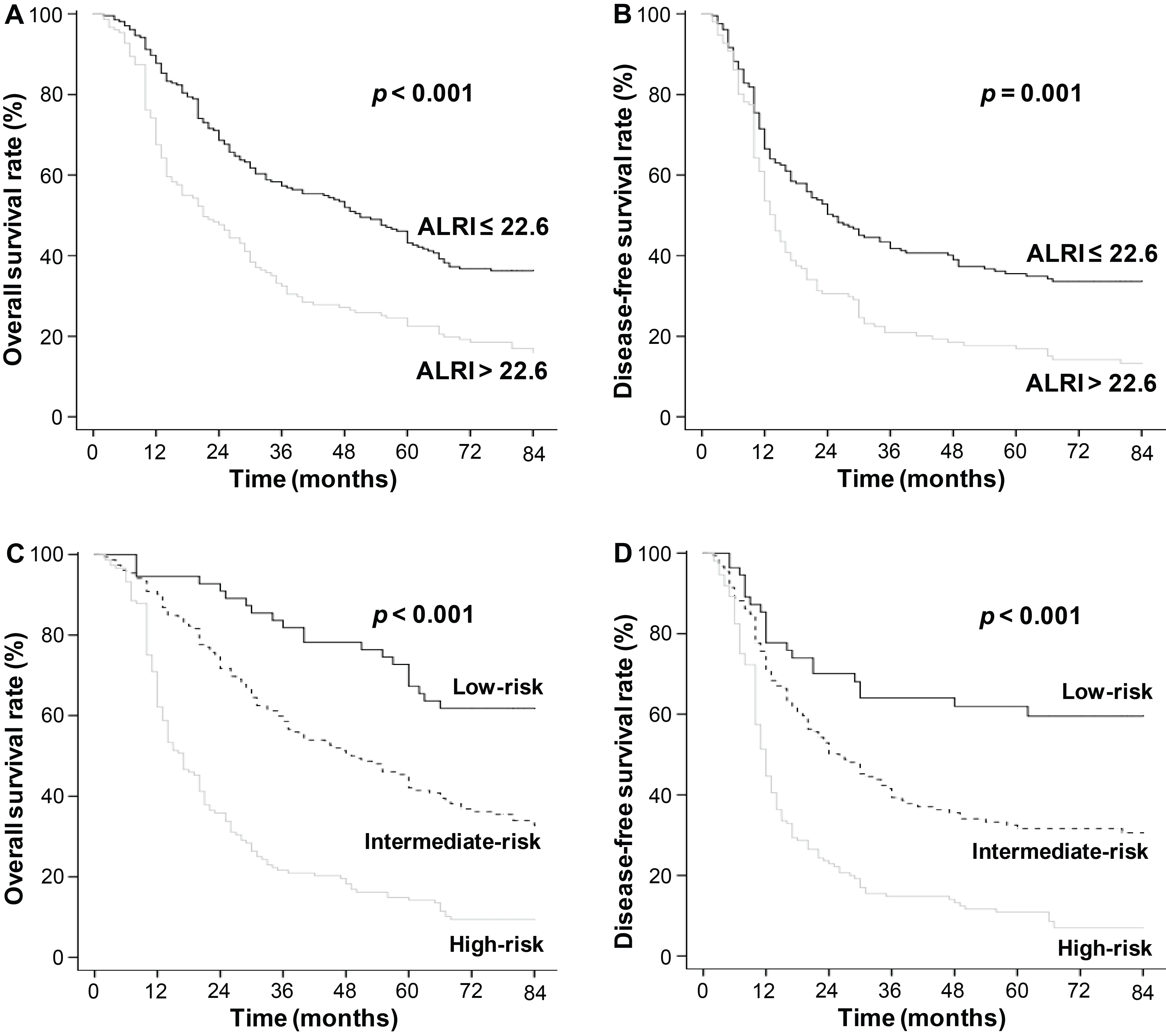


Fig S4. The OS and DFS curves in the validation cohort. Kaplan-Meier survival analyses suggested that HCC patients with ALRI > 22.6 had shorter OS and DFS (A and B). The black line refers to ALRI ≤ 22.6 and the gray line: ALRI > 22.6. Kaplan-Meier survival analyses of HCC patients in different risk groups (C and D). The black line refers to low-risk group, the dotted line: intermediate-risk group and the gray line: high-risk group.
