# Supplementary material for: A Novel Post-Operative ALRI Model Accurately Predicts Clinical Outcomes of Resected Hepatocellular Carcinoma Patients
Source: Front Oncol. 2021 Jul 6;11:665497. doi: 10.3389/fonc.2021.665497 (PMC8290124; doi:10.3389/fonc.2021.665497)
Supplement: Supplementary file 2 [file Table_1.doc]

**Table S1.** Univariate and multivariate Cox regression analyses of the [clinicopathologic](../../../../C:/Program%20Files%20(x86)/Youdao/Dict/6.3.69.8341/resultui/frame/javascript:void(0)%3B)

**characteristics for OS and DFS in validation cohort with HCC.**

| Variable | Univariate analysis | | | Multivariate analysis | | |
| --- | --- | --- | --- | --- | --- | --- |
|  | HR | 95% CI | *p* value | HR | 95% CI | *p* value |
| **Overall survival** |  |  |  |  |  |  |
| Sex (male *vs.* female) | 1.406 | 0.937-2.110 | 0.100 |  |  |  |
| Age, yeas (≤ 55 *vs.* > 55) | 1.234 | 0.948-1.608 | 0.118 |  |  |  |
| HBsAg (positive *vs.* negative) | 1.152 | 0.839-1.581 | 0.382 |  |  |  |
| Tumor size, cm (> 6 *vs.* ≤ 6) | 2.663 | 2.087-3.450 | < 0.001 | 2.105 | 1.619-2.887 | < 0.001 |
| Tumor number (multiple *vs.* single) | 1.829 | 1.409-2.371 | < 0.001 |  |  |  |
| TNM stage (III *vs.* I-II) | 2.537 | 2.003-3.339 | < 0.001 | 1.745 | 1.303-2.335 | < 0.001 |
| Recurrence: absent/present (n) | 1.120 | 0.875-1.436 | 0.369 |  |  |  |
| Albumin, g/L (≤ 34 *vs.* > 34) | 1.553 | 1.217-1.985 | < 0.001 | 1.440 | 1.117-1.873 | 0.006 |
| [Globulin](javascript:void(0);), g/L (> 33 *vs.* ≤ 33) | 1.161 | 0.909-1.483 | 0.233 |  |  |  |
| ALT, U/L (> 38 *vs.* ≤ 38) | 1.056 | 0.826-1.350 | 0.665 |  |  |  |
| GGT, U/L (> 45 *vs.* ≤ 45) | 1.271 | 1.115-1.779 | 0.005 |  |  |  |
| ALP, U/L (> 90 *vs.* ≤ 90) | 1.246 | 1.083-1.658 | 0.013 |  |  |  |
| AFP, ng/ml (> 20 *vs.* ≤ 20) | 1.050 | 0.787-1.399 | 0.737 |  |  |  |
| ALRI level (> 22.6 *vs.* ≤ 22.6) | 1.880 | 1.469-2.405 | < 0.001 | 1.933 | 1.478-2.527 | < 0.001 |
| **Disease-free survival** |  |  |  |  |  |  |
| Sex (male *vs.* female) | 1.474 | 0.982-2.212 | 0.061 |  |  |  |
| Age, yeas (≤ 55 *vs.* > 55) | 1.127 | 0.865-1.466 | 0.237 |  |  |  |
| HBsAg (positive *vs.* negative) | 1.245 | 0.907-1.709 | 0.175 |  |  |  |
| Tumor size, cm (> 6 *vs.* ≤ 6) | 2.427 | 1.901-3.105 | < 0.001 | 1.861 | 1.431-2.685 | < 0.001 |
| Tumor number (multiple *vs.* single) | 1.733 | 1.336-2.249 | < 0.001 |  |  |  |
| TNM stage (III *vs.* I-II) | 2.318 | 1.826-3.004 | < 0.001 | 1.569 | 1.166-2.111 | 0.003 |
| Albumin, g/L (≤ 34 *vs.* > 34) | 1.490 | 1.165-1.904 | 0.001 | 1.327 | 1.019-1.737 | 0.035 |
| [Globulin](javascript:void(0);), g/L (> 33 *vs.* ≤ 33) | 1.179 | 0.923-1.507 | 0.187 |  |  |  |
| ALT, U/L (> 38 *vs.* ≤ 38) | 1.101 | 0.861-1.407 | 0.448 |  |  |  |
| GGT, U/L (> 45 *vs.* ≤ 45) | 1.258 | 1.097-1.596 | 0.009 |  |  |  |
| ALP, U/L (> 90 *vs.* ≤ 90) | 1.213 | 1.017-1.563 | 0.030 |  |  |  |
| AFP, ng/ml (> 20 *vs.* ≤ 20) | 1.071 | 0.803-1.427 | 0.640 |  |  |  |
| ALRI level (> 22.6 *vs.* ≤ 22.6) | 1.715 | 1.338-2.186 | < 0.001 | 1.701 | 1.305-2.218 | < 0.001 |

**Abbreviations:** HR, hazard ratio; CI, confidence interval; HBsAg, hepatitis B surface antigen; TNM, tumor-node-metastasis; ALT, alanine aminotransferase; AST, aspartate aminotransferase; GGT, Gamma-glutamyl transpeptidase; ALP, alkaline phosphatase; AFP, alpha-fetoprotein; ALRI, aspartate aminotransferase to lymphocyte ratio index.
